# Class similarity network for coding and long non-coding RNA classification

**DOI:** 10.1186/s12859-021-04517-6

**Published:** 2021-12-20

**Authors:** Yu Zhang, Yahui Long, Chee Keong Kwoh

**Affiliations:** 1 School of Computer Science and Engineering, Nanyang Technological University, 50 Nanyang Avenue, Singapore, 639798 Singapore; 2 Wellcome Trust – Medical Research Council Cambridge Stem Cell Institute, Cambridge, CB2 0AW UK; 3 College of Computer Science and Electronic Engineering, Hunan University, Changsha, 410000 China

**Keywords:** Long non-coding RNA, mRNA, CNN, Siamese Neural Network

## Abstract

**Background:**

Long non-coding RNAs (lncRNAs) play significant roles in varieties of physiological and pathological processes.The premise of the lncRNA functional study is that the lncRNAs are identified correctly. Recently, deep learning method like convolutional neural network (CNN) has been successfully applied to identify the lncRNAs. However, the traditional CNN considers little relationships among samples via an indirect way.

**Results:**

Inspired by the Siamese Neural Network (SNN), here we propose a novel network named Class Similarity Network in coding RNA and lncRNA classification. Class Similarity Network considers more relationships among input samples in a direct way. It focuses on exploring the potential relationships between input samples and samples from both the same class and the different classes. To achieve this, Class Similarity Network trains the parameters specific to each class to obtain the high-level features and represents the general similarity to each class in a node. The comparison results on the validation dataset under the same conditions illustrate the superiority of our Class Similarity Network to the baseline CNN. Besides, our method performs effectively and achieves state-of-the-art performances on two test datasets.

**Conclusions:**

We construct Class Similarity Network in coding RNA and lncRNA classification, which is shown to work effectively on two different datasets by achieving accuracy, precision, and F1-score as 98.43%, 0.9247, 0.9374, and 97.54%, 0.9990, 0.9860, respectively.

**Supplementary Information:**

The online version contains supplementary material available at 10.1186/s12859-021-04517-6.

## Background

Long non-coding RNAs (lncRNAs) are a kind of transcribed RNA molecule with lengths longer than 200 nucleotides that do not encode proteins [1]. The recent advances in RNA sequencing technologies have attracted wide attention of researchers to lncRNAs. The lncRNAs are reported to play crucial roles in a variety of biological processes, such as epigenetic modification, chromatin remodeling, and gene transcription [2–4]. Moreover, lncRNAs are also proved to closely related to diverse diseases like cancer [5], Alzheimer’s disease [6], *etc*.

However, only a few of lncRNAs have been well-characterized functionally [7]. To better understand the lncRNA function, the lncRNAs need to be identified correctly. Therefore, rapid and accurate in silico methods are needed to help to distinguish coding RNAs (mRNAs) and lncRNAs.

A variety of prediction tools have been developed to classify mRNAs and lncRNAs. CPC (Coding Potential Calculator) is an alignment-based approach developed on Support Vector Machine (SVM) model [8], but it relies heavily on the previously used dataset. Later on alignment-free methods have been developed to overcome the disadvantages of alignment-base methods. Alignment-free methods only use sequence intrinsic information. For example, CPAT (Coding Potential Assessment Tool) is built with the logistic regression model [9], and LncFinder [10] and CPPred [11] are based on the SVM model. Recently, deep learning has also been used in distinguishing mRNAs and lncRNAs, and it is reported to outperform those traditional machine learning models. For example, DeepLNC [12] and RNAsamba [13] are built with the deep neural network (DNN), mRNN (mRNA RNN) is trained with recurrent neural network (RNN) [14], LncRNAnet is developed on the combination of RNN and convolutional neural network (CNN) [15], and DeepCPP [16] are trained on CNN.

When using CNN to train the model, the parameters would be adjusted according to the cost function batch by batch, and epoch by epoch. Therefore, the relationships among input samples are only considered from the cost function indirectly when training CNN. Hence one research question is that, can we improve the CNN model performance by taking more relationship among input samples into consideration in a direct way during the training process?

Inspired by the Siamese Neural Network (SNN) which focuses on learning embeddings in the deeper layer to place the same classes close together [17], here, we propose a novel network named Class Similarity Network to classify mRNAs and lncRNAs by taking more relationships among samples into consideration. Specifically, Class Similarity Network is composed of three modules: Class Similarity Measurement module, Fully Connected module, and Decision module. The Class Similarity Measurement module measures the differences of high-level features between each input sample and samples from both the same class and the different class separately. The highly individualized filters will be trained for each class to facilitate the parameter training on later steps. The Fully Connected module learns the weights and biases from different dense branches and integrates the information to a similarity node for each class. The Decision module concatenates the nodes that represent the similarities for different classes to output the prediction. We note that the Class Similarity Network could achieve higher average accuracy than that of the baseline CNN model on our validation dataset under the same conditions. Besides, the high AUC (Area Under the Curve) values of ROC (Receiver Operating Characteristic) and PRC (Precision-Recall Curve) on two test datasets, i.e. 0.9945 and 0.9981 for ROC, and 0.9990 and 0.9858 for PRC, respectively, as well as the extensive comparisons with state-of-the-art methods on two test datasets demonstrate the effectiveness of our Class Similarity Network.

## Results

### Model hyper-parameters and implementation

The model is implemented by Keras backend in Tensorflow. To determine the number of convolution layers *l* and the hyper-parameters, we test different choices of value for each parameter and record the average Acc (Accuracy), Sp (Specificity), and Sn (Sensitivity) for three times running on validation dataset in Additional file 1: Fig. S1.a-c. Each time we only change the value for one parameter while keeping the value of other parameters unchanged. The value achieving the best Acc after 30 epochs for that parameter is chosen to construct the model.

We determine to use 2 convolution layers and set the kernel size as 2 and stride size as 1 for both layers. We use *adamax* as the optimizer with default parameters reported in its original work [18]. Because the input sets $\mathbf{X} _{pos}^{'}$ and $\mathbf{X} _{neg}^{'}$ can be different when training different models, our final model is an ensemble one by adding the prediction scores of three individual models. Each individual model is trained with 60 epochs (Additional file 1: Fig. S1.d) with batch size 256. The prediction label of the ensemble model is determined according to where the larger probability located in positive or negative class. The complete network structure and parameters are shown in Additional file 1: Fig. S2.

### Influences of $\mathbf{X} _{pos}^{'}$ and $\mathbf{X} _{neg}^{'}$

**Fig. 1:**
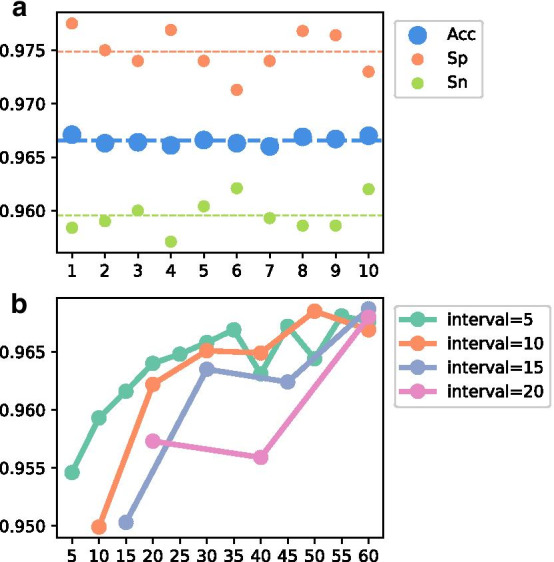
The influences of $\mathbf {X}_{pos}^{'}$ and $\mathbf {X}_{neg}^{'}$. a. The Acc, Sp, and Sn on validation dataset achieved by 10 different sets of $\mathbf {X}_{pos}^{'}$ and $\mathbf {X}_{neg}^{'}$ (x-axis), the average value is marked with the dash line. b. the Acc on validation dataset achieved by changing $\mathbf {X}_{pos}^{'}$ and $\mathbf {X}_{neg}^{'}$ during training with *n* intervals ($n=5,10,15,20$), x-axis: training epoch

The two input sets of the Class Similarity Network during training process, i.e., $\mathbf{X} _{pos}^{'}$ and $\mathbf{X} _{neg}^{'}$, are randomly resampled from the original training positive and negative sets (see Methods). To see if the choice of $\mathbf{X} _{pos}^{'}$ and $\mathbf{X} _{neg}^{'}$ would cause impact influences on the predictions, we randomly generate them 10 times to obtain different input training pairs and record their performances on our validation dataset (Fig. 1a). Besides, we also change the $\mathbf{X} _{pos}^{'}$ and $\mathbf{X} _{neg}^{'}$ during training process with *n* epoch intervals to see whether such practice could improve the prediction performances on the validation dataset, where $n=5,10,15,20$ (Fig. 1b).

**Fig. 2:**
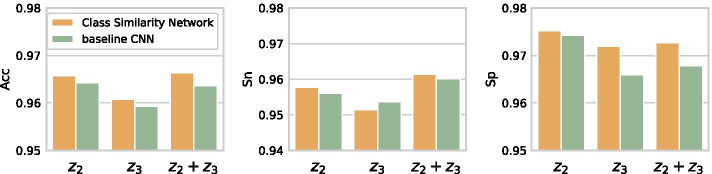
The comparison between Class Similarity Network and baseline CNN model with different dense layers. Evaluation criteria (from left to right) are Acc, Sn, and Sp, where $z_{2}$ represents going through two dense layers: from dropping out 20% data, to 128 neurons with ReLU activation function, and to decision nodes with Sigmoid activation function; $z_{3}$ represents going through one dense layer: from dropping out 50% data to decision nodes with Sigmoid activation function; $z_{2}+z_{3}$ represents the combination of $z_{2}$ and $z_{3}$

**Fig. 3:**
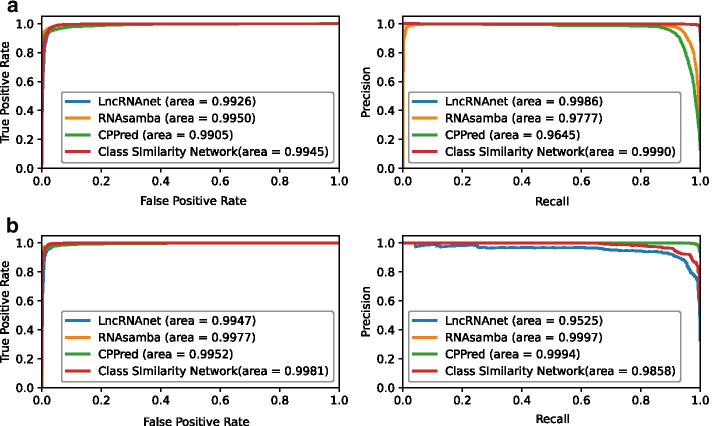
The ROC (left) and PRC (right) curves for different methods on two test datasets. **a** Test dataset I, and **b** Test dataset II

**Fig. 4:**
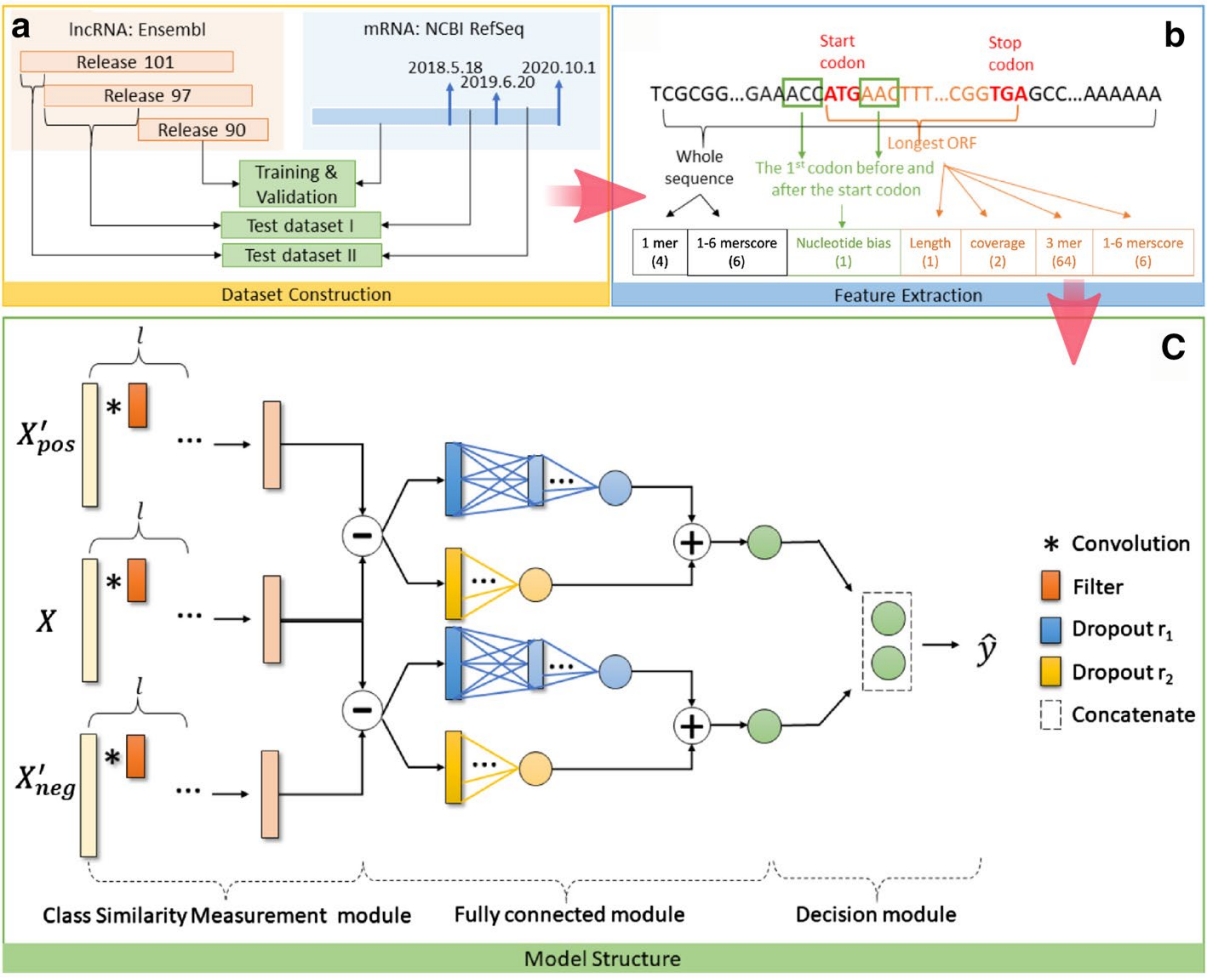
The flow chart. **a** The dataset construction. **b** The feature extraction. **c** An overview of the proposed Class Similarity Network. It contains three modules: Class Similarity Measurement module, Fully Connected module, and Decision module

We note that the overall Acc with 10 different sets of $\mathbf{X} _{pos}^{'}$ and $\mathbf{X} _{neg}^{'}$ range from 96.60 to 96.71%. The small variation indicates the robustness of the Class Similarity Network. Furthermore, the highest Acc with different intervals in Fig. 1b are not always achieved at the last epoch and are not always larger than the Acc achieved without changing $\mathbf{X} _{pos}^{'}$ and $\mathbf{X} _{neg}^{'}$ during training process in Fig. 1a. Therefore, the choice of $\mathbf{X} _{pos}^{'}$ and $\mathbf{X} _{neg}^{'}$ would not cause impact influence on the Class Similarity Network, which demonstrates the model stability.

### Comparison with baseline SNN and CNN

We compare our newly proposed Class Similarity Network with the baseline SNN on our validation dataset. We employ the Euclidean distance metric to construct the SNN model, the layers and parameters for constructing the SNN model are kept in consistent with the Class Similarity Network model (Additional file 1: Fig. S3). We train the SNN model with 60 epochs. The SNN model performs relatively poor on identifying the mRNA samples and it only achieves Acc as 69.61%, which is much lower than that of the Class Similarity Network model whose Acc is 96.36% (Additional file 1: Table S1).

We then compare our newly proposed Class Similarity Network with the baseline CNN on our validation dataset with different dense layers to evaluate the performance of both the Class Similarity Measurement module and the Fully Connected module. Three types of dense layers are adopted, including: (1) dropout 20% input, connected to 128 neurons with ReLU activation function, and then connected with Sigmoid activation function, (2) dropout 50% input, connected with Sigmoid activation function, and (3) the combination of (1) and (2). We use $z_{2}$, $z_{3}$ and $z_{2}+z_{3}$ to represent the above three types of dense layers, respectively, to be consistent to the description in Methods. The average results of 5 times running are shown in Fig. 2.

The values of the Acc and Sp achieved by our Class Similarity Network are all higher than that of the baseline CNN in all three types of dense layers, which indicates the superiority of our method to the baseline CNN. Besides, the results on the validation dataset of our Class Similarity Network show that combining $z_{2}$ and $z_{3}$ could improve the overall Acc and reduce the gap between Sn and Sp in the lncRNA and mRNA classification problem.

### Comparison with state-of-the-art methods

We compare our Class Similarity Network with other recently developed state-of-the-art methods, including mRNN [14], lncRNAnet [15], LncFinder [10], RNAsamba [13], CPPred [11], and DeepCPP [16]. All these methods are developed from 2018 onward. LncFinder and CPPred use SVM model. Other methods use diverse deep learning architectures such as CNN, DNN, RNN, or their combinations, where the deep learning architectures have been verified to play important roles in a variety of lncRNA-related predictions. For example, deep belief network has been applied to identify the lncRNA and predict lncRNA-protein interaction [19]; DNN has been used to predict lncRNA subcellular localization [20] and promoters of coding RNA and lncRNA [21]; more complex deep learning structures have been adopted to predict lncRNA-disease associations [22, 23]. We summarize the features used in the methods for comparisons in this work, as shown in Table 1. The performances for different approaches on two test datasets are recorded in Table 2 and Table 3, respectively, where Sp and Sn represent the Acc of lncRNA and mRNA separately, the *w10u5* model in mRNN and normal model in DeepCPP are used for comparison here.

**Table 1:** The features used in different methods

Feature \Method	mRNN	LncRNAnet	RNAsamba	LncFinder	CPPred	DeepCPP	Class Similarity Network
Maximum ORF length			$\checkmark$		$\checkmark$	$\checkmark$	$\checkmark$
ORF coverage					$\checkmark$	$\checkmark$	$\checkmark$
Nucleotide bias						$\checkmark$	$\checkmark$
k-mer			$\checkmark$			$\checkmark$	$\checkmark$
k-merscore				$\checkmark$	$\checkmark$	$\checkmark$	$\checkmark$
Fikett score					$\checkmark$	$\checkmark$	
Sequence encoding	$\checkmark$ $^{1}$	$\checkmark$ $^{2}$	$\checkmark$ $^{3}$				
Other				$\checkmark$ $^{4}$	$\checkmark$ $^{5}$		

$^{\textsf {1}}$Linear embedding, $^{\textsf {2}}$One-hot, $^{\textsf {3}}$Numeric representations, $^{\textsf {4}}$Euclidean-distance, Logarithm-distance, secondary structural related features, and physicochemical related features, and $^{\textsf {5}}$composition, transition, distribution, ORF integrity, and Isoelectric point, Grand average of hydropathicity, and estimation of the stability of a predicted peptide

**Table 2:** The performances of different methods on test dataset I

Method	Sp (%)	Sn (%)	Acc (%)	Pre	F1-score
mRNN	95.22	96.50	95.38	0.7399	0.8376
LncRNAnet	97.83	95.79	97.57	0.8616	0.9070
RNAsamba	94.06	**98.16**	94.56	0.6996	0.8169
LncFinder	96.03	96.21	96.05	0.7735	0.8576
CPPred	96.02	95.46	95.95	0.7718	0.8534
DeepCPP	${\underline{98.44}}$	${\underline{96.47}}$	${\underline{98.19}}$	${\underline{0.8969}}$	${\underline{0.9294}}$
Class Similarity Network	**98.91**	95.05	**98.43**	**0.9247**	**0.9374**

Bold: the best result, underline: the second best

**Table 3:** The performances of different methods on test dataset II

Method	Sp (%)	Sn (%)	Acc (%)	Pre	F1-score
mRNN	95.33	97.29	97.06	0.9938	0.9832
LncRNAnet	97.85	96.71	96.84	0.9971	0.9819
RNAsamba	94.14	**98.82**	**98.28**	0.9923	**0.9902**
LncFinder	96.77	96.57	96.60	0.9957	0.9805
CPPred	97.37	97.15	97.17	0.9965	0.9838
DeepCPP	**1**	22.73	31.63	**1**	0.3704
Class Similarity Network	${\underline{99.29}}$	${\underline{97.31}}$	${\underline{97.54}}$	${\underline{0.9990}}$	${\underline{0.9860}}$

Bold: the best result, underline: the second best

**Table 4:** The name and size of four datasets used in this work

Dataset name	Number of lncRNA	Number of mRNA
Training	18146	33359
Validation	7150	8557
Test dataset I	23915	3373
Test dataset II	836	6419

In our Class Similarity Network, the amount of lncRNA sample is only about half of the mRNA in the training. But our model achieves the highest and the second highest value of Sp in test dataset I and test dataset II separately. It improves upon other methods by more than 0.47% and 1.44% of Sp on two test datasets, respectively. And also, our model achieves the best Acc, Pre, and F1-score in test dataset I, and the second best Sn, Acc, Pre, and F1-score in test dataset II.

Although the best Sp and Pre (Precision) in test dataset II are achieved by DeepCPP with value of 1, DeepCPP performs poorly in identifying mRNA in test dataset II. Additionally, RNAsamba performs better than our method in predicting mRNA on both two test datasets, and it achieves the best overall Acc on test datset II. However, the good performance of RNAsamba can be attributed to its large input feature sizes. The number of features used by RNAsamba is 2757, but we only use 84 features. Moreover, even if RNAsamba achieves the best Acc in test dataset II, our model leads to higher value of Pre than it. The large number of correctly predicted mRNA samples and the much smaller number of wrongly predicted lncRNA samples contribute to the high Pre value in our model. Besides, when we compare RNAsamba with our method via the McNemar test on the union of test dataset I and II (Additional file 1: Fig. S4, where the McNemar test on two test dataset separately are also shown), we obtain $\chi ^{2}=588.438$ and *p*-$value=0$, which indicates that our method is superior to RNAsamba.

We also plot the ROC curves and PRC on two test datasets for those approaches which return the prediction scores, as shown in Fig. 3. Specifically, test dataset I has much more lncRNA samples ($lncRNA:mRNA\approx 7.09:1$) and test dataset II has much more mRNA samples ($lncRNA:mRNA\approx 1:7.68$). Therefore, the ROC curves and PRC could give us an inference of prediction approaches on potentially imbalance datasets.

Although varied in ratios of different classes, our Class Similarity Network performs good and stable on two test datasets. The AUC of ROC curves are 0.9945 and 0.9981, and the AUC of PRC are 0.9990 and 0.9858, respectively. Our method achieves the best AUC of PRC on test dataset I and the best AUC of ROC on test dataset II. Although RNAsamba achieves the best AUC of ROC on test dataset I and the best AUC of PRC on test dataset II, its AUC of PRC on test dataset I is only 0.9777.

## Discussion

LncRNAs play significant roles in complex pathological and physiological processes. However, the similar properties shared by mRNAs and lncRNAs such as splicing, poly(A) tails, and comparable sequence lengths [24] pose challenges to the identification of lncRNAs from mRNAs. If the use of novel neural network structure could improve the classification accuracy between mRNAs and lncRNAs remains to be explored.

Inspired by SNN, we propose a novel network named Class Similarity Network to classify mRNAs and lncRNAs by considering the relationships among input samples in a direct way. The Class Similarity Measurement module measures the differences of high-level features between input sample and samples in both positive and negative classes with specific filters targeting at each classes. The Fully Connected module learns parameters from different dense branches to integrate similarity information of each class. And the Decision module concatenates the nodes to make the prediction.

We change the selection of input positive and negative sets of the Class Similarity Network and compare it with baseline SNN and CNN using validation dataset. The small variation and the good performances on prediction results show the robustness and the effectiveness of the Class Similarity Network.

We also compare the Class Similarity Network with other machine learning-based state-of-the-art models developed in recent years on different datasets. Class Similarity Network achieves overall accuracy as 98.43% and 97.54% on two test datasets and performs better than other methods in most cases.

However, one limitation for Class Similarity Network in mRNA and lncRNA classification is that it outputs high prediction accuracy of lncRNA but a relatively low accuracy of mRNA. Therefore, strategies should be explored to further improve the prediction accuracy of mRNA. Besides, in case that a large bias exists between training and new test dataset, an alternative representation of entry vectors for each class, i.e. $\mathbf{X} _{pos}^{'}$ and $\mathbf{X} _{neg}^{'}$, should be explored to minimise the prediction errors.

## Conclusions

In this work, we propose Class Similarity Network for mRNA and lncRNA classification by considering the relationships among input samples in a direct way. We compare our approach with baseline models and other recently developed machine learning-based in silico tools on validation dataset and two test datasets. The high prediction results and their small variations illustrate the effectiveness and robustness of the Class Similarity Network in the classification between mRNAs and lncRNAs. We expect that the Class Similarity Network could provide insights and references to characterize the lncRNAs and understand their functions.

## Methods

### Datasets

Four datasets were used in this work, including a training dataset, a validation dataset, and two test datasets. To construct the datasets, we first downloaded the *Human training dataset* and the *Human test dataset* from work of [11] as our training dataset and validation dataset separately. The noncoding RNAs in these two datasets were from Ensembl release 90 [25] (ftp://ftp.ensembl.org/pub/release-90/fasta/homo_sapiens/ncrna/) and the mRNAs were from NCBI RefSeq [26]. Next we downloaded the *New human test dataset* from work of [16] as our first independent test dataset, denoted as test dataset I. The noncoding RNAs in test dataset I were from Ensembl release 97 [25] but not overlapped with release 90, and the mRNAs were from NCBI RefSeq [26] released after 18th May 2018. Besides, we constructed the second independent test dataset, namely test dataset II. We collected the noncoding RNAs from Ensembl release 101 [25] and removed the samples which were overlapped with release 97. And we collected the mRNAs from NCBI RefSeq [26] released between 20th Jun. 2019 and 1st Oct. 2020. The search limitations for all mRNAs in above datasets remained the same as: *“Homo sapiens”[Organism] AND srcdb_refseq_known[prop] AND biomol_mrna[prop]*. We then filtered the above noncoding RNA datasets by only keeping the lncRNAs according to the transcript biotype annotation provided by the Ensembl database. Moreover, the sequences which contain letter other than *’ATGC’* were also removed. The final data amount for each dataset is listed in Table 4 and the dataset construction process is briefly demonstrated in Fig. 4a.

### Features

The feature representation methods we used in this work including: maximum ORF (open reading frame) length and coverage [9], nucleotide bias [16], k-mer, and k-merscore [27].

The longest ORF is often considered to be the coding region [28], hence the features related to the longest ORF are popular to be used in distinguishing mRNAs and lncRNAs. Maximum ORF length feature represents the length of the longest ORF in each RNA sequence, which is denoted as $l_{maxORF}$. Maximum ORF coverage feature is obtained by dividing $l_{maxORF}$ by the length of whole sequence $l_{RNA}$. Because some lncRNAs do not have complete ORF, we represent the maximum ORF coverage for such samples as $[0,\frac{l_{maxORF}}{l_{RNA}}]$ while represent those with complete ORF as $[\frac{l_{maxORF}}{l_{RNA}},0]$. Nucleotide bias feature measures the information around start codon, as studies show that nucleotide around start codon can affect the regulation of translation initiation [29, 30]. Nucleotide bias feature is calculated as

1 $$\begin{aligned} \begin{aligned} nucleotide \ bias = \frac{1}{6}\sum _{i\in \{-3,-2,-1,4,5,6\}}log\frac{p_{mRNA}(x_{i})}{p_{lncRNA}(x_{i})},\\ x\in \{A,C,G,T\} \end{aligned} \end{aligned}$$

where $p(x_{i})$ denotes the frequency of nucleotide *x* at position *i* in training data, and the set of $\{-3,-2,-1,4,5,6\}$ refers to the positions of the first codon before and after the start codon. k-mer feature counts the frequency of *k* neighboring nucleotide into a vector of length $4^{k}$. Here we count the 1-mer feature of the whole sequence and the 3-mer feature of the longest ORF sequence. k-merscore feature represents a relative k-mer bias, its calculation is similar to nucleotide bias:

2 $$\begin{aligned} kmer \,\, score=\frac{1}{4^{k}}\sum _{i=1}^{4^{k}}log\frac{M_{mRNA}(K_{i})}{M_{lncRNA}(K_{i})} \end{aligned}$$

where $M_{mRNA}(K_{i})$ and $M_{lncRNA}(K_{i})$ represents the mean value of k-mer composition for mRNA and lncRNA training data. Here we calculate the k-merscore feature with $k=1-6$ for both the whole sequence and the longest ORF sequence. The feature extraction process is illustrated in Fig. 4b. After applying the above feature representation methods, each RNA sequence would be converted to a fix-length vector with length of 84 (Maximum ORF length (1) and coverage (2), nucleotide bias (1), 1-mer for whole sequence ($4^{1}$), 3-mer for the longest ORF sequence ($4^{3}$), and k-merscore feature with $k=1-6$ for both whole and the longest ORF sequences (6+6)).

### Model structure

We propose Class Similarity Network to classify mRNAs and lncRNAs. The Class Similarity Network is developed on the basis of CNN but considers the relationships among input samples in a direct way. It employs the similar concept of SNN [17] to measure the similarity among input samples. But different from SNN, Class Similarity Network introduces loss function to measure the similarities in each channel, which specifically targets to a two-class classification problem: one for positive class and the other one for negative class. In this way, there are two inputs as references but one input to be contrast, hence it is not suitable to train the same parameters and weights for all subnetworks like what SNN does. To solve this, we design the network to learn the proper parameters itself to encode the input samples from different channels. Besides, different dense branches are integrated to measure the similarity to each class simultaneously. As shown in Fig. 4c, the Class Similarity Network comprises three modules: Class Similarity Measurement module, Fully Connected module, and Decision module. The details are described as follows.

#### Class similarity measurement module

We first measure the similarities between each input sample and a random sample from each class via the high-level features learned by the network, hence we name this module as the Class Similarity Measurement Module. Let $\mathbf {X}_{i}\in \mathbb {R}^{d}, i=1,2,...,k$ denotes the input dataset whose sample size is *k* and feature size is *d*. In the training step of a two classes classification problem, we have a positive dataset $\mathbf{X} _{pos}$ and a negative dataset $\mathbf{X} _{neg}$, where $\mathbf{X} _{pos}\cup \mathbf{X} _{neg}=\mathbf{X}$ and $\mathbf{X} _{pos}\cap \mathbf{X} _{neg}=\emptyset$. We randomly resample from $\mathbf{X} _{pos}$ and $\mathbf{X} _{neg}$ with size of *k* separately and obtain two new sets $\mathbf{X} _{pos}^{'}$ and $\mathbf{X} _{neg}^{'}$. As shown in Fig. 4c, the Class Similarity Measurement Block has three inputs: $\mathbf{X} _{pos}^{'}$, $\mathbf{X}$, and $\mathbf{X} _{neg}^{'}$, the samples in each of them will go though *l* 1D convolutional layers and output as $Conv_{l}(\mathbf{x} _{pos}^{'})$, $Conv_{l}(\mathbf{x} )$, and $Conv_{l}(\mathbf{x} _{neg}^{'})$, respectively. The convolutional layers here convert the raw features to high-level features. With the high-level features, the similarities between input samples and positive samples, and the similarities between input samples and negative samples can be represented as:

3 $$\begin{aligned} f_{ps}(\mathbf{x} )= & {} Conv_{l}(\mathbf{x} )-Conv_{l}(\mathbf{x} _{pos}^{'}) \end{aligned}$$

4 $$\begin{aligned} f_{ns}(\mathbf{x} )= & {} Conv_{l}(\mathbf{x} )-Conv_{l}(\mathbf{x} _{neg}^{'}) \end{aligned}$$

The selections of $\mathbf{X} _{pos}^{'}$ and $\mathbf{X} _{neg}^{'}$ in the test process are different from that of the training. Rather than using the randomly resampled vectors, we use the mean value of $\mathbf{X} _{pos}$ and $\mathbf{X} _{neg}$ from training. Such practice can avoid the potential slightly different prediction results caused by using different resampled datasets.

#### Fully connected module

Taken the $\mathbf{x} _{ps}=f_{ps}(\mathbf{x })$ and $\mathbf{x} _{ns}=f_{ns}(\mathbf{x })$ obtained from Class Similarity Measurement module as the inputs, the Fully Connected module converts each of them to a single value which represents the similarity with positive or negative samples. We represent $\mathbf{x} _{ps}$ with dropout ratio $r_{1}$ as $\mathbf {x}_{ps}^{r_{1}}$ and with dropout ratio $r_{2}$ as $\mathbf {x}_{ps}^{r_{2}}$. From Fig. 4c, we have three dense layers in the Fully Connected module, where $z_{2}$ is obtained from $\mathbf {x}_{ps}^{r_{1}}$ going through two dense layers via $\mathbf {z_{1}}$, and $z_{3}$ is obtained from $\mathbf {x}_{ps}^{r_{2}}$ with one dense layer. The $\mathbf {z_{1}}$, $z_{2}$ and $z_{3}$ are obtained as follows:

5 $$\begin{aligned} \mathbf {z_{1}}= & {} \sigma _{1}\left( \mathbf {W}_{1j_{1}}^{T}\mathbf {x}_{ps}^{r_{1}}+\mathbf {b}_{1j_{1}}\right) \end{aligned}$$

6 $$\begin{aligned} z_{2}= & {} \sigma _{2}\left( \mathbf {W}_{2j_{2}}^{T}\mathbf {z_{1}}+\mathbf {b}_{2j_{2}}\right) \end{aligned}$$

7 $$\begin{aligned} z_{3}= & {} \sigma _{3}\left( \mathbf {W}_{3j_{3}}^{T}\mathbf {x}_{ps}^{r_{2}}+\mathbf {b}_{3j_{3}}\right) \end{aligned}$$

where $\mathbf {W}_{j}$ denotes the weight matrix, $\mathbf {b}_{j}$ denotes the bias vector, and $\sigma (\cdot )$ denotes the activation function. Here we take $\sigma _{1}$ as the ReLU activation function, and take $\sigma _{2}$ and $\sigma _{3}$ as the Sigmoid activation function, and we set the dropout ratio $r_{1}$ and $r_{2}$ as 0.2 and 0.5 separately. Therefore, the positive similarity node is represented as:

8 $$\begin{aligned} y_{ps}=z_{2}+z_{3} \end{aligned}$$

Similarly, the negative similarity node $y_{ns}$ can be obtained by taking $\mathbf{x} _{ns}$ as the input.

#### Decision module

For each input sample, the predicted target $\hat{\mathbf{y }}$ is obtained by concatenating the positive similarity node and the negative similarity node as $\hat{\mathbf{y }}=[y_{ps},y_{ns}]$. Given the target $\mathbf{y}$, our goal is to minimize $\left\| \mathbf {y}-\mathbf {\hat{y}} \right\|$. If the positive target is represented as $\mathbf{y} =[1,0]$ and the negative target is represented as $\mathbf{y} =[0,1]$, the values of $y_{ps}$ and $y_{ns}$ represent the similarity to positive and negative samples separately; if the positive target is represented as $\mathbf{y} =[0,1]$ and the negative target is represented as $\mathbf{y} =[1,0]$, the values of $y_{ps}$ and $y_{ns}$ represent the difference to positive and negative samples separately. Here we use Mean squared error as the loss function, hence our goal is to learn parameters $\theta$ such that:

9 $$\begin{aligned} arg \ minimise_{\theta } \ \frac{\sum _{i}^{N}\left( y_{i}-\hat{y}_{\theta | i}\right) ^{2}}{N} \end{aligned}$$

#### Why class similarity network?

Let *C* denotes the cost function, *z* denotes the output of the dense layer, and *a* denotes the input of the dense layer. In neural networks, the forward pass will calculate the output of the *l*th dense layer as:

10 $$\begin{aligned} z^{l}=\sigma \left( \mathbf {\omega } ^{l}\mathbf {a}^{l-1}+\mathbf {b}^{l}\right) \end{aligned}$$

The weight ${\varvec{\omega }}$ and bias *b* will be updated at the backpropagation step as:

11 $$\begin{aligned} \omega _{jk}^{l}= & {} \omega _{jk}^{l}-\eta \frac{\partial C}{\partial \omega _{jk}^{l}} \end{aligned}$$

12 $$\begin{aligned} b _{jk}^{l}= & {} b _{jk}^{l}-\eta \frac{\partial C}{\partial b_{jk}^{l}} \end{aligned}$$

with learning rate $\eta$. Therefore, the input samples are only related to each other through the cost function and their relationships are only considered in an indirect way. However, in our newly proposed Class Similarity network, the input of the dense layer actually is the differences of high-level features between two samples, i.e. $\Delta \mathbf {a}$, and $C \sim \mathbf {\omega } \Delta \mathbf {a}+\mathbf {b}$. In this way, the differences between two input samples are amplified, and the relationships between different input samples are taken into consideration in a direct way when training the network.

Besides, Class Similarity network introduces filters specified to each class and subtracts the high-level features rather than training the differences of raw features directly. Such practice could help the network to find the proper input values for parameter adjustment in Fully Connected module.

### Evaluation metrics

We use the criteria of Accuracy (Acc), Sensitivity (Sn), Specificity (Sp), Precision (Pre), and F1-score to evaluate different prediction methods, which are calculated as follows:

13 $$\begin{aligned} Sp= & {} \frac{TN}{TN+FP} \end{aligned}$$

14 $$\begin{aligned} Sn= & {} \frac{TP}{TP+FN} \end{aligned}$$

15 $$\begin{aligned} Acc= & {} \frac{TP+TN}{TP+FN+TN+FP} \end{aligned}$$

16 $$\begin{aligned} Pre= & {} \frac{TP}{TP+FP} \end{aligned}$$

17 $$\begin{aligned} F1\text {-}score= & {} \frac{2\times Pre\times Sn}{Pre+Sn} \end{aligned}$$

where TP, FN, TN and FP denote the numbers of true positive, false negative, true negative, and false positive, respectively. Besides, the McNemar test is further adopted to compare the models, which is implemented by using the *mcnemar* function in python package *statsmodels.stats.contingency_tables* with continuity corrected $\chi ^{2}$ distribution.

## Supplementary Information

**Additional file 1.** Supplementary materials (Supplementary Figures S1–S4, Supplementary Tables S1).

## Data Availability

The codes for this work are freely available at: https://github.com/yuuuuzhang/Class-Similarity-Network.

## Abbreviations

AUC: Area under the curve
ROC: Receiver operating characteristic
PRC: Precision–recall curve
Acc: Accuracy
Sn: Sensitivity
Sp: Specificity
Pre: Precision
CNN: Convolutional Neural Network
SNN: Siamese Neural Network
lncRNA: Long non-coding RNA
mRNA: Coding RNA
CPC: Coding potential calculator
SVM: Support vector machine
CPAT: Coding Potential Assessment Tool
DNN: Deep Neural Network
mRNN: mRNA RNN
RNN: Recurrent Neural Network
ORF: Open reading frame
