# Supplementary material for: Class similarity network for coding and long non-coding RNA classification
Source: BMC Bioinformatics. 2021 Dec 20;22:609. doi: 10.1186/s12859-021-04517-6 (PMC8691036; doi:10.1186/s12859-021-04517-6)
Supplement: Supplementary file 1 — Additional file 1. Supplementary materials (Supplementary Figures S1–S4, Supplementary Tables S1). [file 12859_2021_4517_MOESM1_ESM.docx]

**Supplementary Materials for** Class Similarity Network for Coding and Long Non-coding RNA classification

Yu Zhang^1,2^, Yahui Long^3^ and Chee Keong Kwoh^1,*^

^1^School of Computer Science and Engineering, Nanyang Technological University, 639798, Singapore, ^2^Wellcome Trust – Medical Research Council Cambridge Stem Cell Institute, CB2 0AW, Cambridge, UK, ^3^College of Computer Science and Electronic Engineering, Hunan University, Changsha 410000, China.

* To whom correspondence should be addressed. Tel: +65 6790 6057; Fax: +65 6792 6559; Email: asckkwoh@ntu.edu.sg

Table of Contents

[Fig. S1. Class Similarity Network parameter determination. 2](#_Toc88742361)

[Fig. S2. The complete network structure and parameters for the Class Similarity Model built in this work. 3](#_Toc88742362)

[Fig. S3. The network structure and parameters for the SNN model used for comparison in this work. 4](#_Toc88742363)

[Fig. S4. The McNemar test between Class Similarity Network model and RNAsamba model. 5](#_Toc88742364)

[Table S1. The comparison between Siamese Neural Network (SNN) model and Class Similarity Network model on validation dataset. 5](#_Toc88742365)

# Fig. S1. Class Similarity Network parameter determination. (a) Accuracies achieved with different numbers of convolution layers. (b) Accuracies achieved with different choices of kernel size in two convolution layers (layer1, layer2). (c) Accuracies achieved with different choices of stride size in two convolution layer layers (layer1, layer2). (d) The training and test accuracies achieved with different training epochs, the best test accuracy is marked by dash line.


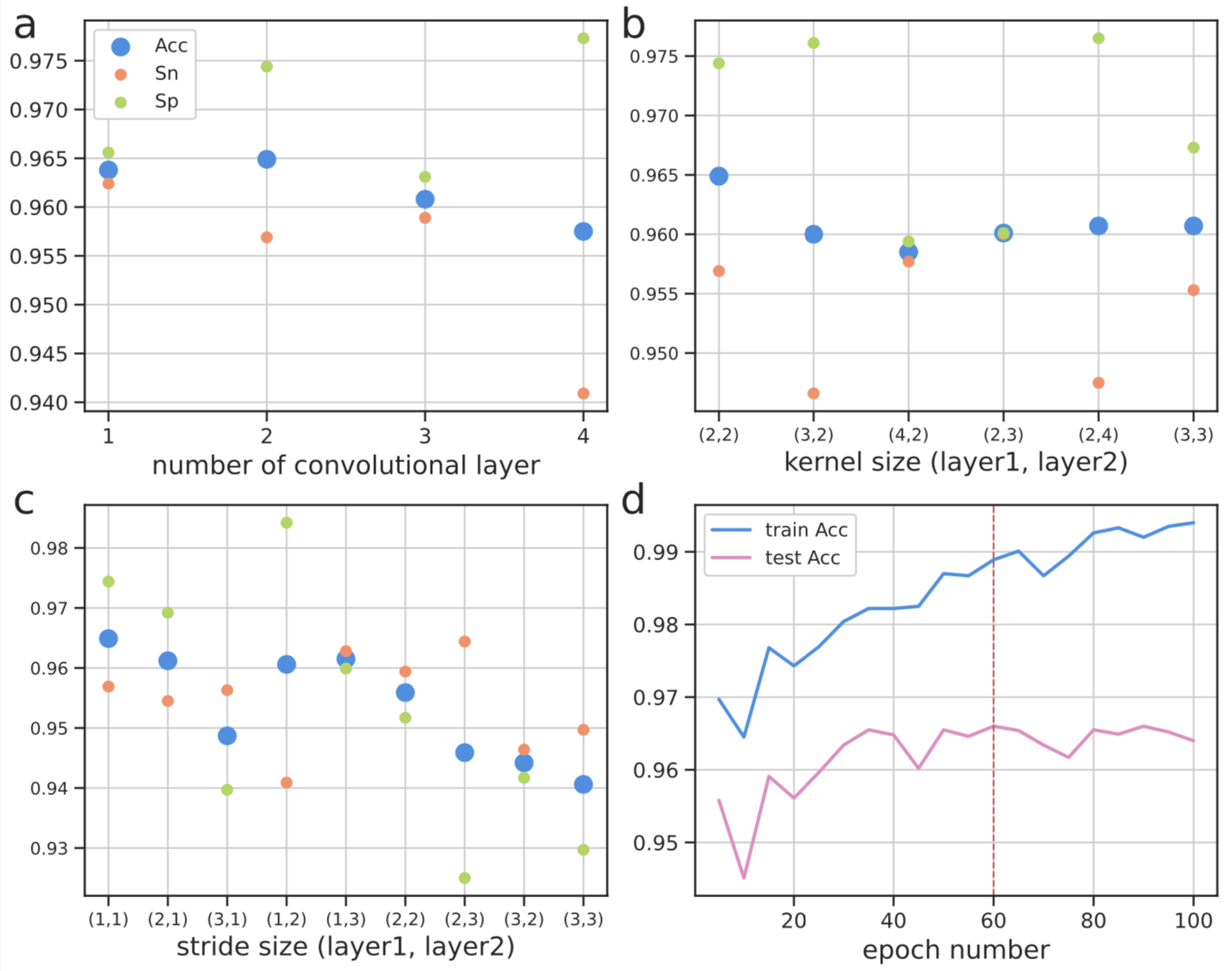


# Fig. S2. The complete network structure and parameters for the Class Similarity Model built in this work.


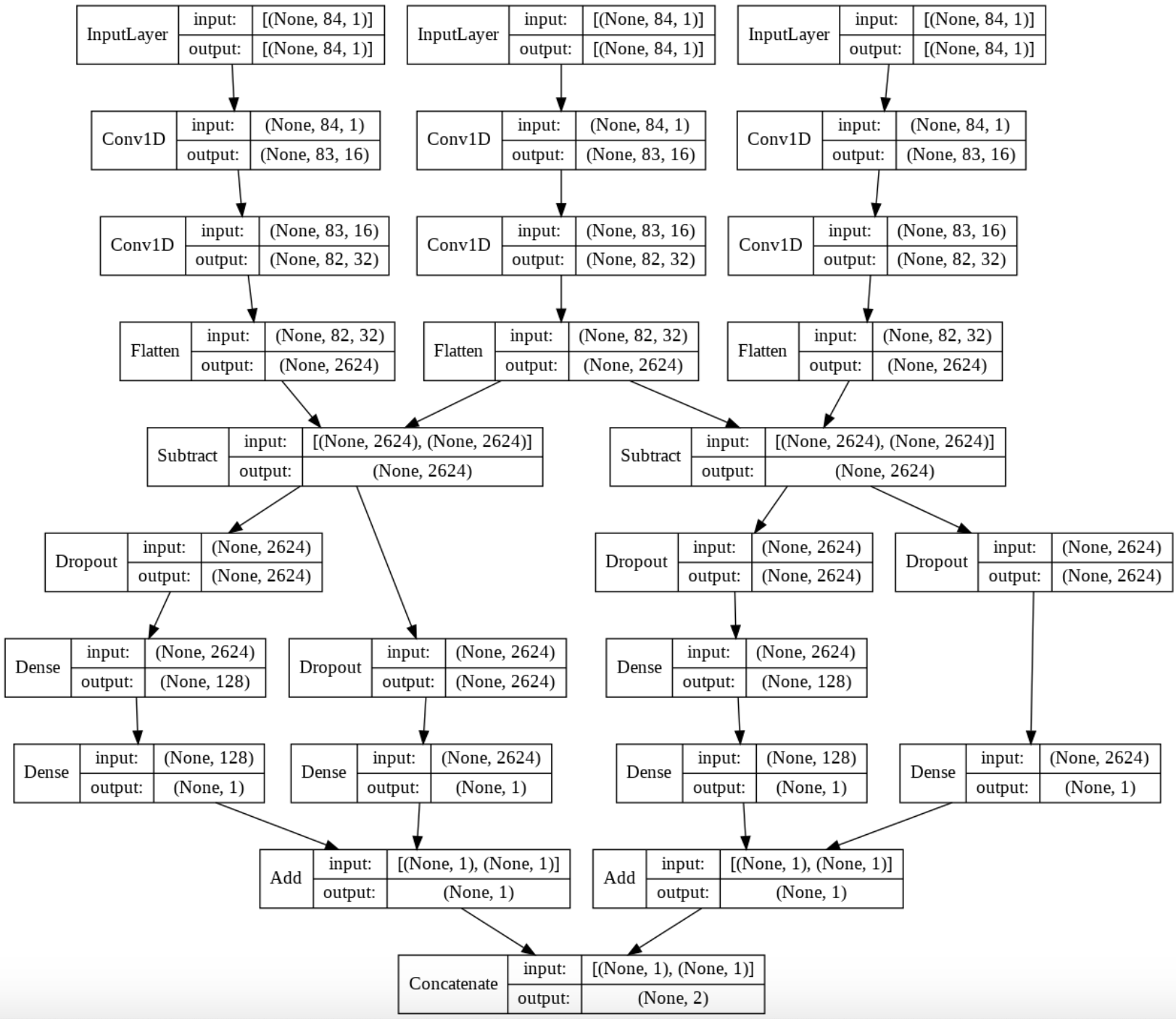


# Fig. S3. The network structure and parameters for the SNN model used for comparison in this work.


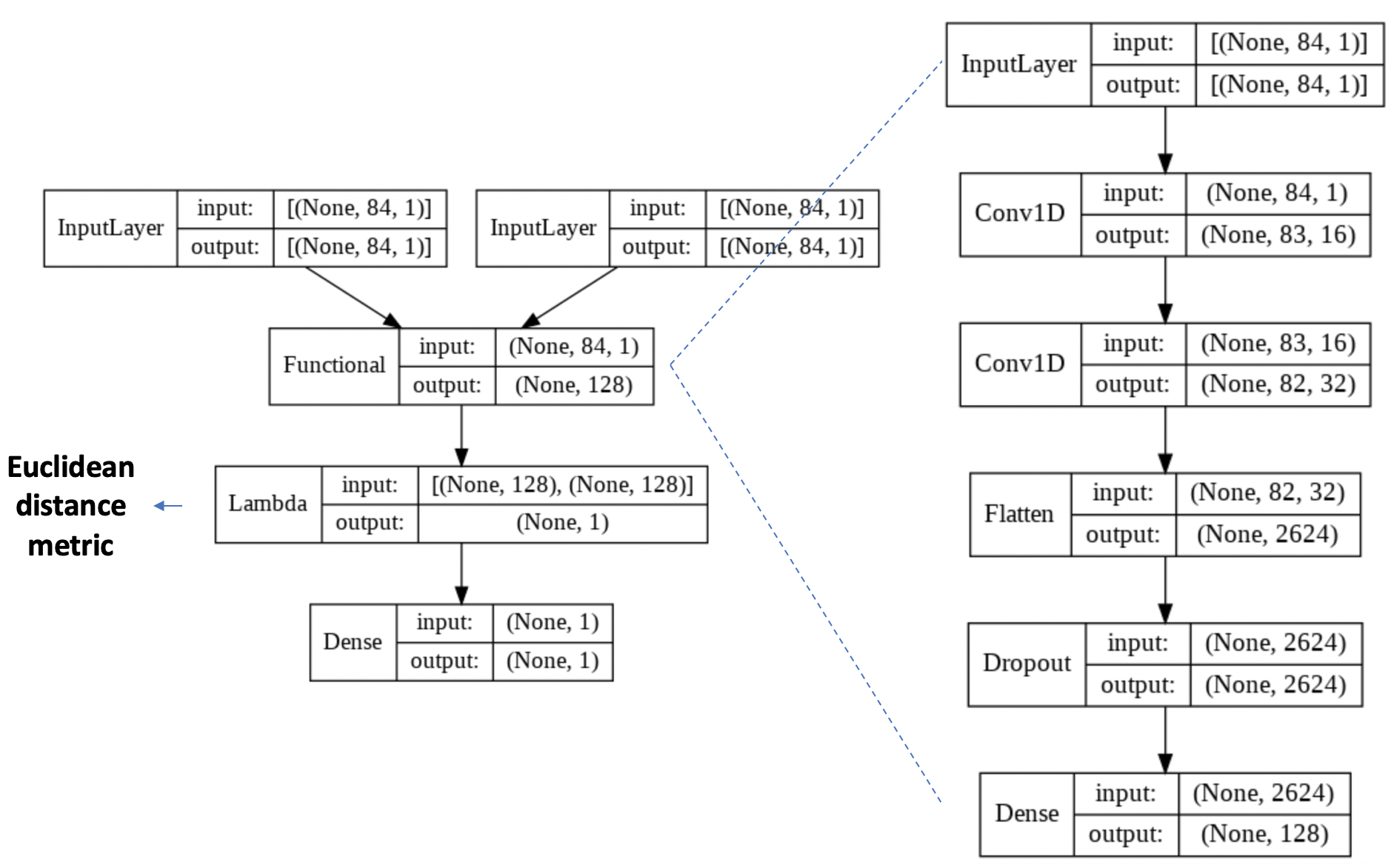


# Fig. S4. The McNemar test between Class Similarity Network model and RNAsamba model on a) the union of the two test datasets, b) test dataset I, and c) test dataset II.

a.


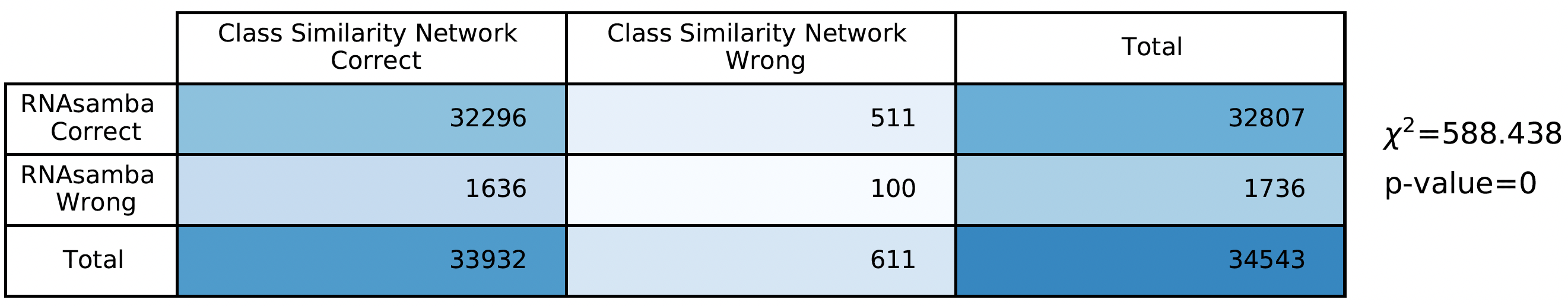


b.


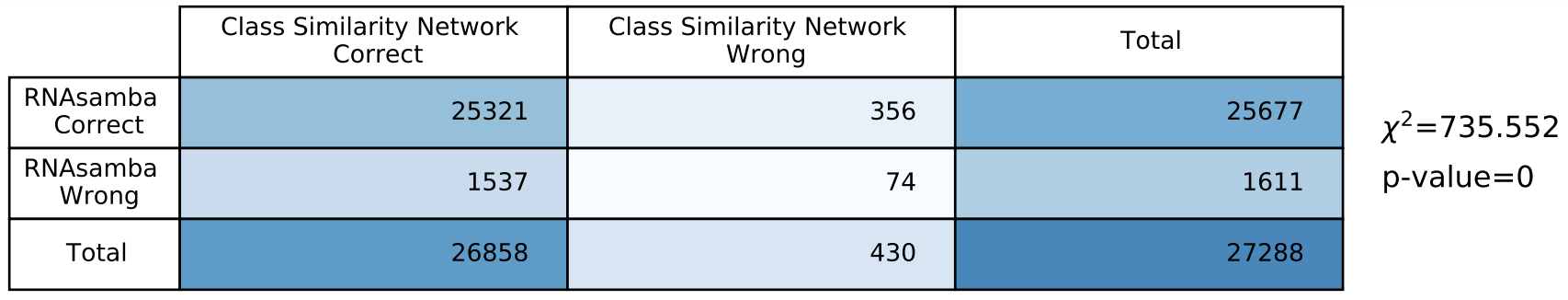


c.


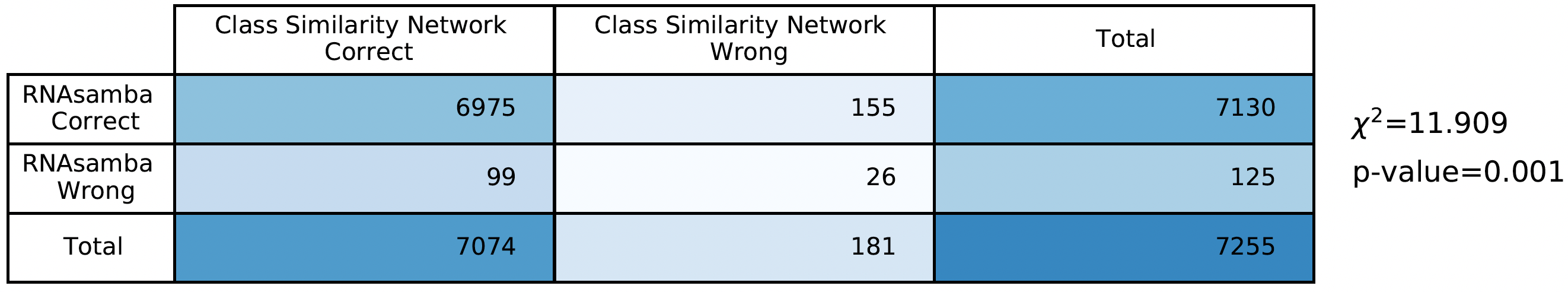


# Table S1. The comparison between Siamese Neural Network (SNN) model and Class Similarity Network model on validation dataset.

|  | Sp (%) | Sn (%) | ACC (%) |
| --- | --- | --- | --- |
| SNN | 95.10 | 48.31 | 69.61 |
| Class Similarity Network | 96.78 | 96.01 | 96.36 |
